# Epigenetic modulation in the treatment of atherosclerotic disease

**DOI:** 10.3389/fgene.2014.00364

**Published:** 2014-10-28

**Authors:** Mikaela M. Byrne, Ross T. Murphy, Anthony W. Ryan

**Affiliations:** ^1^Department of Clinical Medicine and Institute of Molecular Medicine, Trinity Centre for Health Sciences, St. James’s HospitalDublin, Ireland; ^2^Department of Cardiology, St. James’s HospitalDublin, Ireland

**Keywords:** atherosclerosis, cardiovascular disease, epigenetic targeting agents, microRNA, histone deacetylase inhibition, DNA methyltransferase inhibition

## Abstract

Cardiovascular disease is the single largest cause of death in the western world and its incidence is on the rise globally. Atherosclerosis, characterized by the development of atheromatus plaque, can trigger luminal narrowing and upon rupture result in myocardial infarction or ischemic stroke. Epigenetic phenomena are a focus of considerable research interest due to the role they play in gene regulation. Epigenetic mechanisms such as DNA methylation and histone acetylation have been identified as potential drug targets in the treatment of cardiovascular disease. miRNAs are known to play a role in gene silencing, which has been widely investigated in cancer. In comparison, the role they play in cardiovascular disease and plaque rupture is not well understood. Nutritional epigenetic modifiers from dietary components, for instance sulforaphane found in broccoli, have been shown to suppress the pro-inflammatory response through transcription factor activation. This review will discuss current and potential epigenetic therapeutics for the treatment of cardiovascular disease, focusing on the use of miRNAs and dietary supplements such as sulforaphane and protocatechuic aldehyde.

## ATHEROSCLEROSIS

Cardiovascular disease (CVD) is the main cause of death in the western world and its incidence globally is on the rise. It is a multifactorial disease whereby known genetic and environmental effects account for only part of the variability in disease risk (Ordovas and Smith, 2010). Atherosclerosis is the primary cause of CVD and it is characterized by the development of an atheromatus plaque, which triggers luminal narrowing. Plaque rupture can result in myocardial infarction or ischemic stroke.

Atherosclerosis can be defined as a disturbed balance of lipid accumulation, giving rise to chronic inflammation of the arterial wall (Weber and Noels, 2011). Inflammation leads to the recruitment of a variety of immune cells, such as monocytes and T-cells, which lead to further inflammatory signaling and recruitment. This results in the development of a slow progressing lesion and luminal narrowing due to the accumulation of sub-endothelial low density lipoprotein (LDL) and immune cells (Moore and Tabas, 2011).

The initiating phase of plaque development occurs as a result of injury to the endothelium, sources of which include hypercholesterolemia, hypertension, cigarette smoke, diabetes, and obesity; these are often chronic and therefore progress uninterrupted (Ross, 1993). This is followed by the formation of a fatty streak due to the accumulation of foam cells, which are modified macrophages (Hansson and Libby, 2006). A mature plaque develops over time and contains a wider variety of cell types. These have a more complex structure consisting of a necrotic, lipid rich core, and a fibrous cap, the disruption of which leads to the clinical phenotypes mentioned previously (Jonasson et al., 1986). Immune cells are located at the edge of the cap, and upon activation express inflammatory cytokines such as IFN-γ and TNFα which induce vascular cell adhesion molecule-1 (VCAM-1) expression (Cybulsky and Gimbrone, 1991). Elevated levels of circulating cholesterol support CVD and atherosclerosis, which leads to the expression of adhesion molecules and chemokines by endothelial cells, further driving immune infiltration (Hansson and Hermansson, 2011). Atherosclerotic plaques often develop at regions where the arteries branch, where blood flow is non-linear and exerts low shear stress (LSS), which upregulates inflammatory signaling and modulates miRNAs to promote inflammation and monocyte recruitment (Poller et al., 2013; Raitoharju et al., 2013; Webster et al., 2013).

## EPIGENETIC MODIFICATIONS

Epigenetic mechanisms such as DNA methylation, post translational modification (PTM) of histone proteins and RNA mechanisms have been widely investigated in multiple diseases. DNA methylation occurs primarily on CpG dinucleotides and is associated with transcriptional repression through the inhibition of transcription factor binding (De Nigris et al., 2002; Dupont et al., 2009; Burdge and Lillycrop, 2010; Yan et al., 2010). Key PTMs to histone proteins include acetylation, methylation, and phosphorylation. These histone modifications are carried out by various families of enzymes which determine promoter accessibility (De Ruijter et al., 2003; Arrowsmith et al., 2012). RNA mechanisms such as non-coding RNAs (ncRNAs) have the ability to alter gene expression in a variety of ways and play regulatory roles during development, the response to stress and environmental stimuli. ncRNAs can be classified into three groups miRNAs, siRNAs, and LncRNAs (Mercer et al., 2009; Ponting et al., 2009; Kaikkonen et al., 2011). The role these epigenetic modifications play in atherosclerosis and plaque rupture is poorly known in comparison to the well-studied role they play in cancer.

Transgenerational epigenetic inheritance, which refers to the transmission of epigenetic modifications from one generation to the next, can affect the disease susceptibility of offspring (Kilpinen and Dermitzakis, 2012; Skinner et al., 2012). The mechanism behind these effects is not understood (Daxinger and Whitelaw, 2010). However, various epidemiological and population studies have identified transgenerational effects, including modified cardiovascular risk, due to changes in food supply (Lalande, 1996; Kaati et al., 2002; Painter et al., 2008; Maloyan et al., 2013; Bygren et al., 2014; Dominguez-Salas et al., 2014).

Epigenetics can be defined as “The study of mitotically and/or meiotically heritable changes in gene function that cannot be explained by changes in DNA sequence” (Bird, 2002). Here we deal with three well-studied epigenetic mechanisms (DNA methylation, histone modifications, and ncRNAs), which have been the focus of therapeutic intervention in CVD, through targeted drug delivery or dietary supplementation.

### DNA METHYLATION

Animal models have been used to connect DNA methylation/epigenetic modifications and CVD, further suggesting the critical role of DNA methylation in the development of atherosclerosis and CVD (Baccarelli et al., 2010a; Bruneau, 2010; Shirodkar and Marsden, 2011). Studies suggest that altered patterns in methylation correlate with CVD (Castro et al., 2006; Kim et al., 2007, 2010; Chen et al., 2010; McNeil et al., 2011; Soriano-Tárraga et al., 2014; van Kampen et al., 2014; Yamada et al., 2014). Research carried out on genomic DNA isolated from human atherosclerotic lesions, has shown hypomethylation (Sharma et al., 2008). Hypermethylation of atherosclerotic lesions and the promoter region of genes associated with atherosclerosis has also been identified in various genes, e.g. superoxide dismutase, estrogen receptor alpha, endothelial nitric oxide synthase, thus preventing their transcription (Castro et al., 2006; Libby et al., 2010; Jia et al., 2013; Zaina et al., 2014). Methylation of the estrogen receptor B-gene has also been noted to contribute to atherosclerotic progression and is largely associated with aging (Kim et al., 2007).

### HISTONE POST-TRANSLATIONAL MODIFICATIONS

A variety of PTMs have been identified to date. Key PTMs include acetylation, methylation and phosphorylation. These histone modifications are carried out by various families of enzymes which determine promoter accessibility (Arrowsmith et al., 2012). PTMs are often due to the integration of environmental cues at a cellular level and therefore have important roles in diseases related to lifestyle, diet and early life exposure. A number of studies clearly support a link between PTMs and atherosclerotic plaque vulnerability (Stein and Matter, 2011; Bleijerveld et al., 2013; Findeisen et al., 2013; Bentzon et al., 2014; Eom and Kook, 2014; Pucci et al., 2014). The repression of type 1 collagen has been identified as a key step in atherogenesis, with a number of studies identifying the modulators of its repression (e.g., Sin3B) and PTMs which may result in its activation and subsequent plaque stabilization (Kong et al., 2009; Weng et al., 2014). HDAC3 is also known to be involved in plaque rupture; it was identified as the sole histone deacetylase (HDAC) upregulated in ruptured lesions. Inhibition of HDAC3 shifts the phenotype of plaque macrophages to anti-inflammatory and reduces lipid accumulation. Targeting this HDAC may decrease the likelihood of plaque rupture (Hoeksema et al., 2014)

### NON-CODING RNA

The regulation of gene expression is influenced by ncRNAs, which have the ability to alter gene expression in a variety of ways and play regulatory roles during development, the response to stress and environmental stimuli. ncRNAs can be classified into three groups; miRNAs, siRNAs, and LncRNAs (Mercer et al., 2009; Ponting et al., 2009; Kaikkonen et al., 2011). miRNAs regulate genes from a different locus at a post transcriptional level, while siRNAs act to silence the locus from which they are derived (Kaikkonen et al., 2011). LncRNAs have multiple roles, their transcription alone can often alter the expression of nearby genes, and they also carry out essential regulatory roles (Wilusz et al., 2009). A number of studies have identified miRNAs and LncRNAs as key regulators in the development of atherosclerosis (Motterle et al., 2012; Holdt et al., 2013; Chen et al., 2014; Hu et al., 2014b; Liu et al., 2014a,b; Vausort et al., 2014).

## EPIGENETIC TARGETING AGENTS

The use of drugs which target the enzymes responsible for epigenetic modifications such as DNA methyl-transferases (DNMTs) and HDACs have considerable therapeutic potential in CVD and other inflammatory disorders. CVD therapeutic targets include protein coding genes that have an already established relevance in CVD (Poller et al., 2013). Drug therapies that work via epigenetic mechanisms are largely unapproved by the FDA at present and those that are mainly target blood related disorders (see **Table 1** below). Dual therapies are also undergoing trails whereby histone deacetylase inhibitor (HDACi) and DMNTs are combined. This is likely to be used in addition to chemotherapy and interferon treatment in cancer (Egger et al., 2004). In some cases, side effects such as toxicity and non-specific gene modulation have limited their use as cancer preventative agents (Zhang et al., 2013).

**Table 1:** Current FDA approved epigenetic drugs.

Agent	Epigenetic mechanism	Application	Year of approval
Vorinostat	HDAC	CTCL	2006
Romidepsin	HDAC	CTCL	2009
5-Azacytidine	DNMT	MDS	2004
Decitabine	DNMT	MDS	2006
Ruxolitinib	JAK1/2	Myelofibrosis	2011

*CTCL, cutaneous T cell lymphoma; MDS, myelodysplastic syndrome.*

## DNA METHYLTRANSFERASE INHIBITION

Findings have highlighted that epigenetic variation and modification are widely implicated in CVD. However, the mechanism and result of these alterations remain widely unresolved. Conflicting results as to the degree of DNA methylation isolated from atherosclerotic lesions and from patient PBLs have been noted (Castro et al., 2006; Sharma et al., 2008; Baccarelli et al., 2010a,b; Chen et al., 2010). Hypermethylation of the promoter regions of genes associated with atherogenesis has also been observed in genes such as superoxide dismutase, further driving inflammation and atherogenesis, implicating methylation as a potential therapeutic target for the future (Buysschaert et al., 2008; Pons et al., 2009; Libby et al., 2010; Lenfant, 2013).

## HDACi

### SULFORAPHANE AND NUTRITIONAL EPIGENETICS

Extensive epidemiological evidence and animal studies suggest that cruciferous vegetables such as broccoli may help prevent and/or delay various inflammatory disorders. Sulforaphane [1-isothiocyanato-4-(methylsulfinyl)-butane] is thought to be principally responsible for the health benefits associated with cruciferous vegetables due to its activity as a histone deacetylase inhibitor (Myzak et al., 2007; Elbarbry and Elrody, 2011). Sulforaphane acts as an indirect antioxidant to induce the expression of several enzymes via epigenetic modifications of the Nrf2 pathway, which leads to the induction of its downstream anti-oxidative stress pathway (Bai et al., 2013; Zhang et al., 2013). Anti-oxidant response element (ARE) binds Nrf2 in the promoter region, resulting in the induction of a series of anti-oxidant, stress/detoxifying enzymes, and proteins, e.g., heme oxygenase-1 (HO-1), NAD(P)H dehydrogenase [quinone] 1 (NQO-1), uridine 5’-diphospho-glucuronosyltransferas (UGT), glutathione S-transferases (GST), ferretin, thioredoxin, thioredoxin reductase 1, and manganese superoxide dismutase (MnSOD) (Zhang et al., 2013). Numerous epidemiological and animal studies support sulforaphane’s role as an anti-oxidant and epigenetic modifier, suggesting that the risk of atherosclerosis and CVD could potentially be reduced by its use (Zakkar et al., 2009; Evans, 2011; Juurlink, 2012; Kim et al., 2012; Miao et al., 2012; Yoo et al., 2013).

Sulforaphane has been shown to aid the suppression of inflammatory mediators such as VCAM-1 in endothelial cells, through its activation of Nrf2 and alters the physiology of vascular and inflammatory cells (Zakkar et al., 2009; Evans, 2011; Miao et al., 2012). High shear stress results in the development of athero-protected sites through activation of Nrf2. LSS does not, however, activate Nrf2 resulting in pro-inflammatory activation (Chen et al., 2009; Zakkar et al., 2009). Pharmacological activation of Nrf2 using sulforaphane has been show to suppress p38 activation, VCAM-1 expression and ROS production in cultured ECs and in wild type mice. Nrf2–/– mice were unaffected by treatment with sulforaphane, indicating that the anti-inflammatory effects of Sulforaphane are Nrf2 dependent (Chen et al., 2009). This implies that inflammation at athero-susceptible regions can be reduced/prevented through sulforaphane mediated activation of Nrf2. Dietary intervention in human subjects has demonstrated that low concentrations of sulforaphane supplements can inhibit HDAC activity (Myzak et al., 2007). To date sulforaphane is also known to inhibit HDAC activity in human colorectal cancer cells (Myzak et al., 2007).

### CURCUMIN

Curcumin also offers great potential as a dietary atheroprotective agent. Curcumin was shown to reduce the extent atherosclerotic lesions and induce changes in the expression of genes involved in cell adhesion, transendothelial migration in APOE–/– mice fed with 0.2% curcumin over 4 months (Coban et al., 2012). Oligomeric and monomeric flavonoid consumption has been demonstrated to modulate the expression of genes associated with CVD (Milenkovic et al., 2014). These studies highlight a new angle for the treatment and prevention of CVD through the use of dietary supplements. Curcumin when packaged with sulforaphane has been suggested as a chemotherapeutic option for the treatment of pancreatic cancer (Jayaraman et al., 2012; Sutaria et al., 2012).

### PROTOCATECHUIC ALDEHYDE

Protocatechuic aldehyde (PA), an established HDACi, is a compound isolated from the aqueous extract of the root of the *Salvia miltiorrhiza* herb. It has been widely used in traditional Chinese medicine to treat various vascular diseases. There is evidence to suggest that PA can inhibit migration and proliferation of vascular smooth muscle cells (Moon et al., 2012). PA has been shown to affect the expression of adhesion molecules in human umbilical vein endothelial cells (HUVECs) stimulated with TNF-α, suggesting that PA inhibits TNF-α stimulated VCAM-1 and intercellular adhesion molecule-1 (ICAM-1) expression in these cells through a mechanism that involves NF-κB and AP-1 (Zhou et al., 2005). The role of PA in myocardial ischemia and reperfusion injury has been investigated to determine its anti-inflammatory effect *in vivo* and its use as a potential therapeutic agent. The use of a rat model suggested that PA could protect the heart from myocardial ischemia and reperfusion injury by reducing myocardial infarct size and the activities of creatine kinase-MB and cardiac troponin (Wei et al., 2013). As with sulforaphane, the above studies of PA suggest the use of dietary supplements which act as HDAC inhibitors to help prevent the development and progression of atherosclerotic plaque.

## miRNA BIOMARKERS AND INTERVENTIONS

Studies are currently investigating a potential link between miRNA expression profiles, plaque development, and rupture. The use of miRNAs in therapy includes organ targeted RNAi using viral vectors or synthetic RNA, and therapeutic strategies on the basis of modulation of miRNA function (Poller et al., 2013). However, challenges remain with issues such as specificity, as the 3′ UTR of a single mRNA can be targeted by multiple miRNAs. miRNAs also exert many different actions dependent on cell type; hence miRNA modulation therapies require precise cellular targeting and suitable delivery methods. For example miR-144-3p has been shown to accelerate plaque formation through the post transcriptional regulation of ABCA1. ABCA1 has a critical role in cellular cholesterol eﬄux and the formation of HDL. Inhibition of ABCA1 through miR-144-3p has been shown in THP-1 cells and in APOE–/– mice to increase inflammatory cytokine secretion and accelerate plaque formation (Hu et al., 2014a). This highlights how essential it is for appropriate miRNA targeting in atherosclerosis as one miRNA can influence multiple pathways. miRNA expression profiles are also likely to differ between atherosclerotic plaques and healthy arteries, and may provide useful markers and targets in atherosclerosis (Wierda et al., 2010; Cipollone et al., 2011; Raitoharju et al., 2011; Hao et al., 2014; Menghini et al., 2014).

Many of the genes down regulated included those involved in the regulation of signal transduction, transcription, and vesicular transport. Those which were up regulated are thought to be involved in the key processes of atherosclerosis (Wierda et al., 2010; Raitoharju et al., 2011).

Cipollone et al. (2011) investigated whether a unique miRNA signature was associated with plaque instability in humans. They identified five miRNAs, miR-100, miR-127, miR-145, miR-133a, and miR-133b which had altered expression levels. Expression of these miRNAs was greater in symptomatic plaques from patients with ischemic stroke, suggesting a role for miRNAs in plaque instability and rupture, but also as predictive biomarkers. Further findings implicate miRNAs such as miR-24 and miR-21 in the regulation of matrix metalloproteinase (MMPs), known to play a part in fibrous cap thinning and in turn plaque rupture (Di Gregoli et al., 2014; Fan et al., 2014). Endothelial miRNAs are likely to be selectively regulated by arterial flow conditions, particularly the combination of pro-atherogenic LSS and oxidized low-density lipoprotein (oxLDL) which induce the upregulation of miR-92a, suggesting the use of a miR-92a antagomir as a potential therapeutic option (Loyer et al., 2014).

The potential future use of miRNAs in diagnosis and as treatment options remains promising, as a recent article highlights the effect of an RNAi drug ALN-PCS on the synthesis of proprotein convertase subtilisin/kexin type 9 (PCSK9), in one of the first successful applications of RNAi therapeutics in a clinical setting (Abifadel et al., 2003; Zhao et al., 2006; Fitzgerald et al., 2014). Treatment resulted in a mean 70% reduction in circulating PCSK9 plasma protein and a mean 40% reduction in LDL cholesterol from baseline relative to placebo (Fitzgerald et al., 2014). Anti miR-133 therapy has also been shown to reduce the progression of atherosclerosis and improve HDL function in Ldlr –/– mice (Rotllan et al., 2013).

Circulating miRNAs have also attracted considerable attention as potential biomarkers, as they can be detected in blood and potentially used for the risk stratification of patients (Deiuliis et al., 2014). Fichtlscherer et al identified four miRNAs which were reduced in patients with CAD compared to healthy controls, miR-126, miR-17, miR-92a, and miR-155. Muscle enriched miRNAs were also found to be more highly expressed in patients with CAD compared to volunteers (Fichtlscherer et al., 2010; Stellos and Dimmeler, 2014). miRNAs are also involved in the regulation of macrophage phenotype alterations from inflammatory to anti-inflammatory, and they can be altered by shear stress (Boettger et al., 2009; Alexy et al., 2014; De Paoli et al., 2014). These studies clearly identify a correlation between miRNA expression and plaque instability. A number of considerations must also be taken into account such as whether the patients were receiving statins or other cholesterol modifying drugs, as this could alter/contribute to the miRNA expression observed by acting as epigenetic modifiers.

## CONCLUSION

Recent trends have seen advances in the progression of epigenetic drugs from bench to bedside, further supporting their importance as key drugs of the future. However many of the therapies targeting atherosclerotic disease remain unavailable for use in a clinical setting. Issues such as delivery of miRNAs to localized areas remain. However, we are now beginning to see the use of nanoparticles to deliver epigenetic inhibitors such as sulforaphane and ALN-PCS. The specificity of epigenetic therapies is also a consideration, as non-specific effects may result in gene activation/inhibition that may further hinder treatment. Unlike cancer, epigenetic therapy for atherosclerotic disease is still in its infancy, yet it represents a topic of great global concern as it is the main cause of death in the western world. Future directions and preventative measures may include the biomarker profiling of patients with high and low risk plaques through the use of circulating miRNAs, leading to considerable economic and health benefits.

## AUTHOR CONTRIBUTIONS

Mikaela M. Byrne, Ross T. Murphy, and Anthony W. Ryan co-authored the manuscript.

## Conflict of Interest Statement

The Guest Associate Editor, Dr. Steven G. Gray, declares that despite having collaborated with the authors in the past 2 years, the review process was handled objectively and no conflict of interest exists. The authors declare that the research was conducted in the absence of any commercial or financial relationships that could be construed as a potential conflict of interest.
